# Expression and function of NKp46 W32R: the human homologous protein of mouse NKp46 W32R (Noé)

**DOI:** 10.1038/srep40944

**Published:** 2017-01-30

**Authors:** Ariella Glasner, Batya Isaacson, Ofer Mandelboim

**Affiliations:** 1 The Lautenberg Center for General and Tumor Immunology, The Hebrew University Hadassah Medical School, Jerusalem, 91120, Israel

## Abstract

Natural killer (NK) cells eradicate infected cells and tumors following the triggering of activating receptors, like the Natural Cytotoxicity Receptors (NCRs), which include NKp30, NKp44 and NKp46. NKp46 is the only NCR expressed in mice (mNKp46), and except for some Innate Lymphoid Cell (ILC) populations (ILC1/3 subsets), its expression is restricted to NK cells. Previously, a mouse named Noé was generated in which a random point mutation (W32R) impaired the cell surface expression of mNKp46. Interestingly, the Noé mice NK cells expressed twice as much of the transcription factor Helios, and displayed general non-NKp46 specific hyperactivity. We recently showed that the mNKp46 W32R (Noé) protein was expressed on the surface of various cells; albeit slowly and unstably, that it is aberrantly glycosylated and accumulates in the ER. Interestingly, the Tryptophan (Trp) residue in position 32 is conserved between humans and mice. Therefore, we studied here the human orthologue protein of mNKp46 W32R, the human NKp46 W32R. We demonstrated that NKp46 W32R is aberrantly glycosylated, accumulates in the ER, and is unstable on the cell surface. Furthermore, we showed that overexpression of NKp46 W32R or Helios resulted in augmented NK cell activation, which may be applied to boost NK activity for therapeutic applications.

NK cells are innate lymphocytes, lately characterized as group I innate lymphoid cells (ILCs). They specialize in the eradication of various pathogens and cancers^1^,^2^,^3^,^4^,^5^,^6^,^7^,^8^,^9^,^10^,^11^,^12^. A balance of signals derived from inhibitory and activating receptors regulates NK cell cytotoxicity^13^,^14^,^15^,^16^,^17^,^18^. NKp46 is an important NK activating receptor. It belongs to the Natural Cytotoxicity Receptor (NCRs) family, which also includes NKp30 and NKp44. Interestingly, NKp46 is the only NCR to have a mouse orthologue, denoted mNKp46^19^. NKp46 directly recognizes the hemagglutinin (HA) proteins of influenza viruses^4^,^5^,^20^, poxviruses^8^ and Newcastle disease viruses^9^. Recently we demonstrated that NKp46 also recognize the EPA 1/6 and 7 proteins of Candida glabrata^21^. NKp46 also recognizes self and tumor ligands, although the identity of these ligands remains mostly unknown.

We have generated an Ncr1 KO mouse, in which the transmembrane and extracellular coding sequences of Ncr1 were replaced by GFP (denoted Ncr1^*gfp/gfp*^). Using these mice, we and others have demonstrated that NKp46 takes part in many immunological activities^5^,^7^,^10^,^22^,^23^,^24^,^25^. In 2012, a mutant mouse was generated using random N-ethyl-N-nitrosourea (ENU) mutagenesis, which caused a point mutation in position 32 of the activating receptor mNKp46. This mutation led to the substitution of a Trp residue to an Arg residue (W32R) (the mouse was named Noé^26^). In this mouse, mNKp46 was not detected on the cell surface, and a general hyper-, mNKp46-independent responsiveness against various challenges was observed^26^. In contrast, the Ncr1^*gfp/gfp*^ mice manifested impaired mNKp46-dependent functions^5^. To address these differences we studied the properties of the mNKp46-Noé (W32R) protein. We showed that mNKp46 W32R can be expressed on the cell surface, however unstably, that it functions similarly to WT mNKp46, that it is arrested in the ER, and is aberrantly glycosylated^27^. These findings indicate that the Noé phenotype probably stems from the aberrant expression of mNKp46 W32R rather than from its absence.

Interestingly, the human NKp46 protein also contains Trp in position 32. Therefore, we mutated this residue in mNKp46 and studied the properties of NKp46 W32R.

## Results

### Mouse and human NKp46 W32R proteins are expressed on the cell surface, albeit slowly and unstably

**Figure 1:**
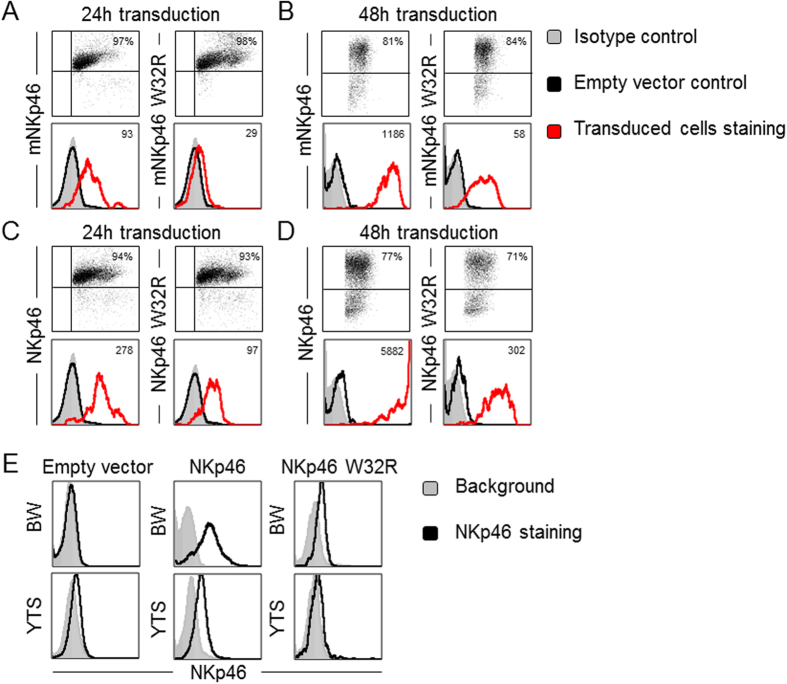
Mouse and human NKp46 W32R proteins are expressed on the cell surface. (**A–D**) FACS plots of HEK293T (293T) cells transduced with an empty vector or the various NKp46 constructs, as indicated. Upper plots: GFP (reporting for the transduction efficiency, percentages are indicated). Lower plots: The gray filled histograms represent the staining of isotype control in the empty vector transduced cells. The black and red line histograms represent staining of the transduced cells with anti mNKp46 (**A,B**) or NKp46 (**C,D**) mAbs. Median Fluoresce Intensity (MFI) of the transduced cells is indicated in each plot. The figure summarizes three independent experiments. (**E**) FACS staining of BW (upper) and YTS (lower) cells transduced with an empty vector and the various constructs, as indicated above each plot. The gray filled histograms represent the staining of isotype control in the empty vector transduced cells; the black line histograms are staining with specific mAbs. The figure summarizes three independent experiments. Throughout the figure, isotype control staining of all other transduced cells was similar to the empty vector control, therefore it are not presented.

To test whether the Trp residue in position 32 of the human NKp46 would affect its activity, we generated a point mutation in human NKp46, replacing Trp with Arg in position 32 (NKp46 W32R). The wild type (WT) and mutated NKp46 constructs were cloned into lentiviral vectors that express GFP, which reports for transduction efficiency. We initially evaluated the expression kinetics of NKp46 W32R in 293T cells and compared it to the WT NKp46 and to WT mNKp46 and mNKp46 W32R. As we previously observed^27^, mNKp46 W32R was expressed in delay on the cell surface, and its expression levels were lower than WT mNKp46 (Fig. 1A and B). Importantly, GFP expression was similar among all transduced cells, indicating that the transduction efficiency was similar (Fig. 1A and B). The same experiments were performed using human NKp46 and NKp46 W32R. Consistent with the expression kinetics of mNKp46 W32R, NKp46 W32R was expressed in delay on the surface and its levels were lower than WT NKp46 (Fig. 1C and D). GFP levels were again similar (Fig. 1C and D), indicating similar transduction efficiencies. Notably, the expression of the mNKp46 and NKp46 mutated proteins, but not the WT proteins, gradually decreased within the following days (data not shown).

We also expressed the WT and mutated NKp46 proteins in the mouse BW cells and human YTS cells. Whereas the WT NKp46 cell surface expression was relatively high, NKp46 W32R was poorly expressed (Fig. 1E), and its expression gradually disappeared from the cell surface (data not shown).

### Characterization of NKp46 W32R properties

**Figure 2:**
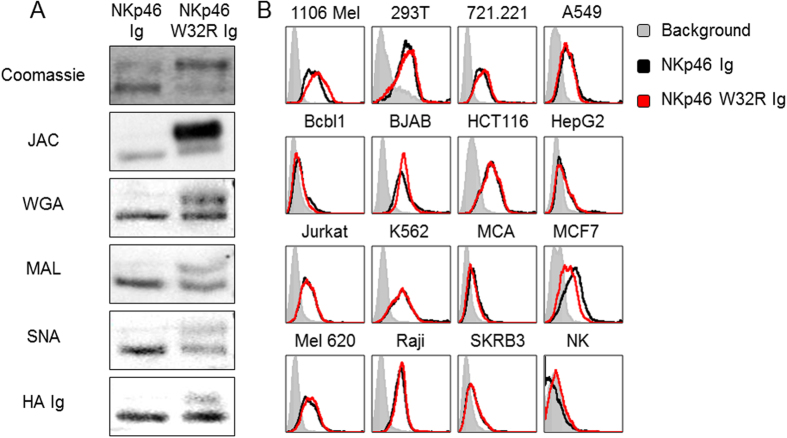
Glycosylations and binding properties of NKp46 and NKp46 W32R fusion proteins. (**A**) Upper: Coomassie staining of the two fusion proteins (5 μg) run on 10% SDS PAGE gel in reducing conditions. Five Lower panels: Western blots performed on the various NKp46 fusion proteins. Staining was performed with the indicated lectins, or biotinylated HA Ig. The figures are representative of three independent experiments. Contrasts were adjusted for clarity. (**B**) FACS staining of various human tumor lines with NKp46 and NKp46 W32R fusion proteins. The filled gray histograms represent staining with the secondary antibody and the black and red line histograms are stainings with the specific fusion proteins. The figure summarizes three independent experiments.

To test whether the glycosylation pattern of NKp46 W32R is impaired, as observed in mNKp46 W32R^27^, we cloned the NKp46 and NKp46 W32R extracellular parts and fused them to human IgG1 (NKp46 Ig and NKp46 W32R Ig respectively). We then expressed these constructs in 293T cells, purified the secreted fusion proteins on a protein G column and ran them on an SDS PAGE gel in reducing conditions (Fig. 2A, upper panel). Both NKp46 Ig and NKp46 W32R Ig displayed two protein bands, however, the staining intensity varied considerably. For the NKp46 Ig the lower band was more intense, while the opposite was observed for NKp46 W32R Ig (Fig. 2A).

We speculated that the two protein bands represent differently glycosylated protein products, and that the W32R mutation leads to an impairment of NKp46 Ig glycosylations. To study this, we performed a Western Blot (WB) with four lectins: Jacalin (JAC) and wheat germ agglutinin (WGA), both of which preferentially bind O-linked glycosylations. Maackia Amurensis lectin II (MAL), which preferentially binds sialic acid residues attached in an α2,3 linkage, and Sambucus Nigra lectin (SNA), which preferentially binds sialic acids attached to a terminal galactose in a α2,6 linkage^28^. All four lectins recognized the lower band of WT NKp46 Ig, while minimal recognition of the upper band was observed (Fig. 2A), indicating that in NKp46 Ig the lower band carries most of the glycosylations. In contrast, all four lectins recognized both upper and lower bands of NKp46 W32R Ig (Fig. 2A).

We have previously demonstrated that NKp46 and NKp44 recognize the influenza virus’s Hemagglutinin (HA) in a sialic acid mediated manner^23^,^27^,^29^,^30^,^31^. Accordingly, we next investigated the HA recognition of NKp46 W32R. We cloned the extracellular part of the HA protein of influenza PR8 and fused it to human IgG1. The chimeric construct was subsequently expressed in 293T cells and purified on a protein G column. We then biotinylated the HA Ig protein, and used it to directly bind the NKp46 and NKp46 W32R fusion proteins in WB assays. HA Ig recognized the lower bands of both NKp46 Ig and NKp46 W32R Ig, and the upper band of NKp46 W32R was also slightly recognized (Fig. 2A).

We also assessed whether NKp46 and NKp46 W32R display congruent binding capabilities of different tumor cell lines. Human NK cells were used as negative control (Fig. 2B). All tested human tumor lines were similarly recognized by NKp46 Ig and NKp46 W32R Ig proteins (Fig. 2B).

### NKp46 W32R is arrested in the ER

**Figure 3:**
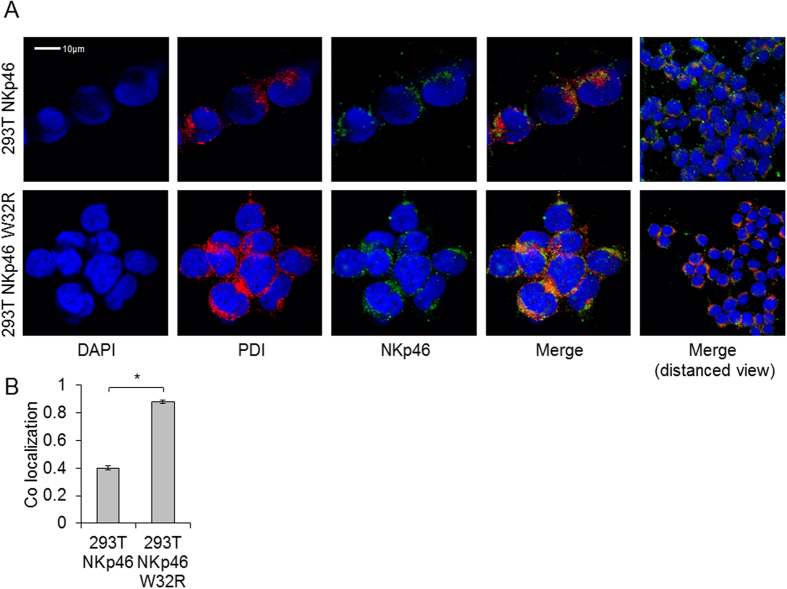
NKp46 W32R proteins are arrested in the ER. (**A**) Confocal microscopy of NKp46 and NKp46 W32R proteins expressed in 293 T cells and stained with anti NKp46, and anti PDI mAbs. The figures are representative of three independent experiments. (**B**) Pearson’s coefficient assessment of the co-localization between NKp46 and PDI. Statistics was performed with at least 30 cells in each correlation. Values are shown as mean ± SEM. *P < 0.05.

Because the NKp46 W32R protein exhibits an altered glycosylation pattern and is poorly expressed on the cell surface, we next studied the intracellular localization of NKp46 and NKp46 W32R. We transduced 293T cells with WT NKp46 and NKp46 W32R constructs and stained the cells for NKp46 along with the ER marker Protein Disulfide Isomerase (PDI) (Fig. 3). Minimal co-localization was observed between the WT NKp46 and PDI. In contrast, NKp46 W32R, which was poorly expressed on the cell surface (Fig. 1), co-localized with PDI (Fig. 3A, quantified in Fig. 3B). These results indicate that the NKp46 W32R protein is arrested in the ER.

### Helios transcript is elevated in NKp46 W32R expressing cells

**Figure 4:**
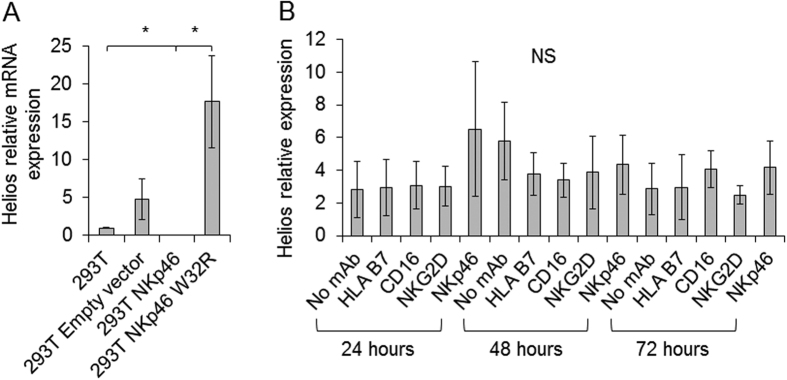
Helios transcript is elevated in NKp46 W32R expressing cells. (**A**) Quantitative PCR for relative expression of the Helios transcript in 293T cells, 293T cells transduced with an empty vector control, NKp46 and NKp46 W32R. The experiment was conducted three times. Values are shown as mean ± SEM. *P < 0.05. (**B**) Quantitative PCR for relative expression of the Helios transcript in primary IL2-activated NK cells following activation with plate bound mAbs. The experiment was conducted three times. Values are shown as mean ± SEM. NS = Non Significant.

It was reported that the Helios transcript was twice as abundant in NK cells in the Noé mice as compared to WT mice^26^. We previously showed that ectopic expression of the mutated mNKp46 W32R protein within the cell leads to increased Helios expression^27^. To test whether overexpression of NKp46 W32R would also lead to increased Helios expression, we transduced 293T cells with NKp46 and NKp46 W32R, and assessed the levels of Helios transcript. As seen in Fig. 4A, an approximate three-fold increase in Helios mRNA levels was observed in 293T cells expressing NKp46 W32R when compared to 293T cells, 293T cells expressing an empty vector control, or 293T cells expressing the WT NKp46. We next asked whether the observed increased Helios expression is related directly to binding of the mutated receptor by a potential intracellular ligand. To address this question, we incubated NK cells with plate bound anti NKp46 mAb, and assayed Helios expression at three time points following incubation. We also were interested to compare NKp46 chronic signaling effect on Helios expression with the signal related to chronic activation of other NK activating receptors (CD16 and NKG2D). As seen in Fig. 4B, Helios levels where unchanged following chronic activation by any of the tested receptors (anti HLAB7 mAb was used as control).

### NKp46, NKp46 W32R and Helios transduced primary human NK cells are activated by influenza and tumor ligands

**Figure 5:**
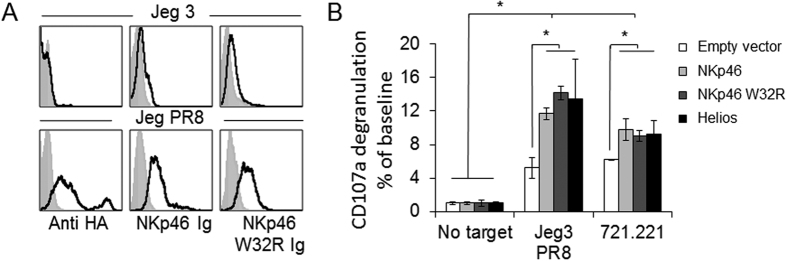
Expression and function of NKp46 and NKp46 W32R in primary human NK cells. (**A**) FACS staining of Jeg3 cells and Jeg3 cells coated with influenza virus with anti HA, NKp46 Ig and NKp46 W32R Ig. The gray filled histograms represent the secondary antibody control, and the black line histograms are stainings with the mAb or fusion proteins. The figure combines data from three independent experiments. (**B**) Primary, activated bulk human NK cells were transduced with the indicated constructs. The various NK transfectants were incubated with the indicated targets and CD107a degranulation (% of baseline) was assessed. The experiment was conducted four times. Values are shown as mean ± SEM. *P < 0.05.

We finally wanted to test whether NKp46 W32R is functional and whether the increased Helios expression observed following NKp46 W32R expression can indeed lead to better NK cell activation. To test this, we generated lentiviral constructs expressing Helios, NKp46 and NKp46 W32R, along with a GFP reporter. We then transduced primary IL2-activated human NK cells with the various constructs, and incubated the transduced NK cells with influenza virus-bound Jeg3 cells and with 721.221 cells. We previously reported that Jeg3 cells do not express NKp46 ligands (35–37). Following incubation with influenza virus, the influenza-bound Jeg3 cells were recognized by an anti-HA mAb, by NKp46 Ig, and by NKp46 W32R Ig (Fig. 5A). We next used the transduced NK cells incubated with the targets, and measured the levels of CD107a on the NK cells. While NK cells incubated with no target showed minimal degranulation, influenza-bound Jeg3 and 721.221 cells caused significant degranulation in NK cells, as indicated by the increased levels of CD107a in NK cells transduced with an empty vector control (Fig. 5B). CD107a degranulation in untransduced NK cells and NK cells transduced with an empty vector control was similar (data not shown). Overexpression of WT NKp46, NKp46 W32R or Helios, resulted in significantly higher levels of CD107a, which was similar in all the transduced NK cells (Fig. 5B).

## Discussion

NKp46 is an important NK activating receptor whose involvement in many immunological conditions was firmly established, using an Ncr1 KO mouse (Ncr1^*gfp/gfp* ^5). Surprisingly, observations to the contrary were made several years ago, using a mouse named Noé. The Noé mouse exhibited a hyperactive, mNKp46-independent phenotype along with elevated Helios levels^26^. To understand the striking differences between the Noé and Ncr1^*gfp/gfp*^mice, we characterized the molecular and functional properties of mNKp46 W32R. We observed that while mNKp46 W32R was mostly arrested in the ER, it could still be expressed on the cell surface, however its expression was unstable^27^. Thus, we proposed that the Noé mouse is not mNKp46 deficient, but rather expresses an aberrant protein, which results in an mNKp46-independent hyper-activation phenotype.

**Figure 6:**
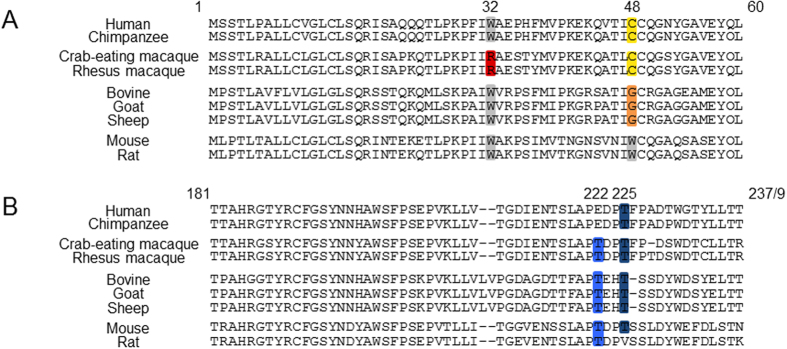
NKp46 multiple sequence alignment. (**A**) Multiple sequence alignment of positions 1-60 of NKp46 proteins, taken from UniProt. Positions 32 and 48 are highlighted in color. (**B**) Multiple sequence alignment of positions 181-237/9 of NKp46 proteins, taken from UniProt. Positions 222 and 225 are highlighted in color.

To investigate whether the properties of mNKp46 W32R are specific only to mNKp46, we introduced the same mutation in the human NKp46, since both mouse and human NKp46 express a Trp residue in position 32 (Fig. 6).

We began our study by expressing NKp46 and NKp46 W32R in mouse and human cells. In agreement with our previous observations^27^, NKp46 W32R was expressed on the cell surface, however, the expression levels were low and it was unstable. To further study the NKp46 W32R properties, we generated fusion proteins of NKp46 and NKp46 W32R. This is a significant finding by itself, since despite the observed impaired expression of the W32R proteins (mouse and human), these mutated proteins could nevertheless be expressed and secreted. We then determined that NKp46 W32R Ig was glycosylated differently from NKp46, using various lectins. Nevertheless, the WT and mutated proteins were found to recognize tumor targets and the influenza virus similarly. Hence, while the dissimilar glycosylation pattern of the WT and mutated proteins affects the proteins’ expression, it does not seem to affect target recognition.

How a single point mutation in one domain of NKp46 can affect the glycosylations of the entire protein is an important question. It was proposed^26^ that the reason why mNKp46 W32R is not expressed on the cell surface is due to a possible disruption in a potential π stacking between W32 and W48. π stacking is a non-covalent interaction, which occurs between aromatic rings, as they contain π bonds^32^,^33^. Interestingly, in the human NKp46 protein, a π stacking interaction cannot occur, as no aromatic rings are present on the side chain of Cys at position 48 (Fig. 6), ruling out the π stacking possibility.

This raises the possibility that maybe the Trp residue in position 32 is essential for the NKp46 activities. However, multiple sequence alignment between the various known sequences of NKp46 revealed that Trp at position 32 is not conserved among different species (Fig. 6A). Human, chimpanzee, bovine, goat, sheep, mouse and rat express Trp in position 32. Interestingly, in macaque monkeys, position 32 is occupied by Arg (Fig. 6A), the same amino acid that is found in mouse or human NKp46 W32R. It is noteworthy that macaque NK cells express normal, functional NKp46^34^. As the structure which can exist only between aromatic rings cannot occur in the NKp46 of most species, it is possible that the W32R mutation in mouse and human interrupts a certain sequence or leads to slower egress to the cell surface of both receptors, which is different in Macaques. Still, it seems that other residues in NKp46 are also involved in its glycosylation as the Macaques NKp46 functions normally^34^. Additionally, in bovine, goat and sheep, position 48 is occupied by the amino acid Glycine (Gly), which is unlikely to form any interactions with the Trp in position 32.

In contrast, as we have recently discovered, the mouse and human glycosylatated residues are conserved^35^. Multiple sequence alignment across the various species revealed that the Thr residues, which may potentially carry O-linked glycosylations, occupy either positions 222, or 225 or both and are conserved across all tested species (Fig. 6B).

It was reported^26^, that mRNA levels of the transcription factor Helios were increased two fold in the NK cells of Noé mouse and this was suggested to lead to hyper-responsiveness. It was also suggested that this might stem from impaired NK cell education *in vivo* in the absence of mNKp46^26^. Interestingly, Helios levels were unchanged in Ncr1^*gfp/gfp*^mice^36^. Previously, we demonstrated that increased Helios expression occurs *in vitro* when mNKp46 W32R is present^27^. Here we demonstrated that it is also true regarding NKp46 W32R. This further supports the notion that the presence of the mutated protein inside the cell results in a general stress response. We currently don’t know why the expression of NKp46 W32R leads to increased Helios expression and NK hyperactivation. As chronic activation by various NK cell receptors did not affect Helios expression in NK cells, we concluded that the aberrant accumulation of proteins in the ER leads to a general stress response that leads to Helios overexpression.

Additionally, we transduced NK cells with Helios, NKp46 and NKp46 W32R and assayed the degranulation of all transduced NK cells when challenged with influenza virus and tumor targets. Interestingly, overexpression of all three constructs resulted in a similar increased NK degranulation, compared to NK cells transduced with an empty vector control. Because NKp46 W32R and Helios transduced cells do not over-express NKp46 (when the NKp46 W32R cells were used, the protein was no longer expressed on the cell surface), these results support the notion that the W32R mutated proteins overexpression in cells causes general hyper-activation, which is probably Helios-dependent.

Taken together, our findings suggest that a W32R mutation in NKp46 results in altered glycosylations, ER arrest and elevated Helios expression. As NK cells overexpressing NKp46 W32R as well as Helios exhibited increased activation, overexpression of Helios might prove a promising option for NK cell therapy.

## Methods

### Cells

The cells used in this study were the mouse BW thymoma, human HEK293T (293T), YTS NK and primary IL2 activated bulk NK cells. The human NK cells used in this study were obtained from the blood of healthy volunteers. The institutional Helsinki committee of Hadassah approved the study (Helsinki number 0030–12-HMO). All handling and preparation of the samples were carried out according to the guidelines and regulations of the Helsinki committee of Hadassah. All subjects provided a written informed consent.

Additional cells used were 1106Mel melanoma cells, 721.221 LCL, A549 lung carcinoma, Bcbl1 body cavity based lymphoma, BJAB B lymphoid leukemia, HCT116 colon colorectal, HepG2 hepatocellular carcinoma, Jeg3 choriocacinoma, Jurkat T cell leukemia, K562 chronic myelogenous leukemia, MCA hepatoma, MCF7 adenocarcinoma, Mel 620 melanoma, Raji Burkitt’s lymphoma and SKBR3 breast cancer.

### NKp46 plasmids, reporter cells, Ig fusion proteins and antibodies

The fusion proteins used in this study were NKp46 Ig, NKp46 W32R Ig and HA Ig. Fusion proteins were generated in 293T cells, as previously described^20^. The W32R single point mutation in NKp46 was generated using the primers:

NKp46 Fw: NotI aat gcggccgc gcc gcc acc atg tct tcc aca ctc cct gcc, NKp46 Rev: MluI aat acgcgttcaaagagtctgtgtgttcagc, NKp46 W32R Fw: cgttcatcagggccgagcc, NKp46 W32R Rev: ggctcggccctgatgaacg.

Staining with all fusion proteins was performed with 5 μg of each (saturating concentration). FACS staining with the fusion proteins was visualized using a conjugated secondary anti-human mAb. For the expression of NKp46 and NKp46 W32R in mouse and human cells, NKp46 and NKp46 W32R constructs were expressed in a lentiviral vector and transduced into cells. The antibodies used for NK cell activation were anti NKp46^20^, anti CD16^37^ anti NKG2D (Biolegend, BLG-320814) and anti HLAB7^38^.

### Western Blotting and lectins

For Western Blotting (WB), NKp46 and NKp46 W32R fusion proteins were run on a 10% SDS PAGE gel, transferred to a nitrocellulose membrane (Tamar, Israel) and blotted with biotin conjugated Jacalin (JAC) lectin, wheat germ agglutinin (WGA) lectin, α2,3 MAL lectin α2,6 SNA lectin (all from Vector labs, Burlingame) and with biotin conjugated HA Ig. The staining was visualized using a streptavidin HRP secondary reagent and EZ ECL substrate (BI, Israel).

### Immunofluorescence

293T cells transduced with NKp46 and NKp46 W32R were grown on glass slides and fixed in 4% PFA. Cells were fixed and permeabilized in cold (−20 °C) methanol and treated with 0.1% triton. Cells were blocked in CAS block (Life Technologies) and incubated with anti-Protein Disulfide Isomerase (PDI) and anti-NKp46. A confocal laser-scanning microscope (Zeiss Axiovert 200 M; Carl Zeiss MicroImaging, Thornwood, New York, USA) was used to analyze the stained slides.

### NK transduction, CD107 degranulation assays and A/Puerto Rico/8/34 H1N1 (PR8) influenza virus infection

NK cells were transduced with lentiviral constructs as previously described^35^. For CD107a degranulation assays, the transduced NK cells were incubated with the targets in a ratio of 1:1 in the presence of 0.1 μg APC conjugated CD107a mAb for 2 hours at 37 °C (Biotest, Kfar Saba, Israel). CD107a levels on the NK cells were determined by flow cytometry. Transduced NK cells were detected by GFP. When influenza virus was used, 1*10^6^ cells in 2 ml complete medium were treated with 20 μl, 1000HU of the PR8 virus over night at 37 °C, 5% CO_2_. Propagation of the human influenza virus A/Puerto Rico/8/34 H1N1 (PR8) was performed as previously described^39^.

### Statistical analysis

Analysis of Variance (ANOVA) was used to identify significant group differences. To assess co-localization, the FV10-ASW version 03.00.01.15 statistical pack was used, followed by Student’s T test, and subsequently comparing the average Pearson coefficient values in at least 30 cells in each group. P < 0.05 was considered significant in all studies.

## Additional Information

**How to cite this article**: Glasner, A. *et al*. Expression and function of NKp46 W32R: the human homologous protein of mouse NKp46 W32R (Noé). *Sci. Rep.* **7**, 40944; doi: 10.1038/srep40944 (2017).

**Publisher's note:** Springer Nature remains neutral with regard to jurisdictional claims in published maps and institutional affiliations.
